# Identification and verification of microRNA signature and key genes in the development of osteosarcoma with lung metastasis

**DOI:** 10.1097/MD.0000000000032258

**Published:** 2022-12-09

**Authors:** Fanjian Meng, Lulu Wang, Guangyu Gao, Jinpeng Chen, Xinghua Wang, Gaochen Wu, Yiqi Miu

**Affiliations:** a Department of Orthopedics, Suzhou Hospital of Integrated Traditional Chinese and Western Medicine, Suzhou, P.R. China; b Department of Oocology, the Second Affiliated Hospital of Soochow University, Suzhou, P.R. China.

**Keywords:** GEO, IHC, microRNA, OS, SLC7A8

## Abstract

Osteosarcoma (OS) is a heterogeneous malignant spindle cell tumor in children under the age of 20. This study aims to research the association between Solute Carrier Family 7 Member 8 (SLC7A8) as well as related genes and OS.

**Method:**

OS and normal samples (GSE38698 and GSE85537) were downloaded from Gene Expression Omnibus dataset. The bioinformatics analysis was performed to distinguish 2 differentially expressed genes, prognostic candidate genes and functional enrichment pathway. Immunohistochemistry and quantitative real-time PCR were utilized for further study.

**Results:**

There were 5 DEMs and 10 differentially expressed genes in cancer tissues compared to normal tissues. According to the km-plot software, ARHGEF3, BSN, PQLC3, and SLC7A8 were significantly related to the overall survival of patients with OS. Furthermore, Multivariate analysis included that SLC7A8 was independent risk factors for OS patients. Furthermore, immunohistochemistry and quantitative real-time PCR outcomes indicated that the expression level of SLC7A8 and hsa-miR-506 was differentially expressed in lung metastasis OS tissues and non-metastasis tissues.

**Conclusion:**

The prognostic model based on the miRNA-mRNA network could provide predictive significance for prognosis of OS patients, which would be worthy of clinical application. Our results concluded that SLC7A8 may play a key role in the development of OS.

## 1. Introduction

Osteosarcoma (OS) is one of the most common types of bone cancer, which usually affects adolescents or young people aged 10 to 30. OS accounts for 2% of all cancer cases in children and adolescents. Compared with other more common cancers such as lung carcinoma and breast carcinoma, this is a very “rare” cancer with a global incidence of 3.4 parts per million per year.^[1–3]^ Although OS is rare in children under the age of 5,^[4]^ the incidence of OS in children aged 5 to 15 is 5.6 parts per million per year.^[2]^ Therefore, OS is more common in adolescence. In the United States, the estimated incidence of OS is 800 to 900 new cases per year, half of which are children and adolescents. OS is also rare in China,^[5]^ and its development trend is almost similar to that in other parts of the world. Although OS is diagnosed early, it can be diagnosed at any age, even at a considerable age. Extraosseous OS is also rare, but the median age of diagnosis is 55.5 years. The metastasis rate in China is higher than that of traditional OS.^[6]^ Treatment of OS needs a lot of methods including many experts, such as primary care doctors, orthopedic oncologists, medical oncologists, radiologists, and pathologists.^[7]^ This cooperation has produced significant benefits. The 5-year survival rate of OS is between 70% and 80%.^[8]^ However, the prognosis of patients with OS needs to be improved. OS is a common metastatic tumor. Its lung metastasis is one of the focuses of future research.^[9–11]^ It should be noted that the 5-year survival rate of patients with metastatic diseases decreased significantly, no more than 10% to 30%.^[12]^

Since the discovery of lin-4 and let-7, the founding members of the microRNA (miRNA) family, hundreds of miRNAs have been found in viruses, plants, and animals by molecular cloning and bioinformatics methods.^[13]^ There are more than 1000 known human miRNAs, which control more than 50% of mammalian protein-coding genes. MiRNAs can be overexpressed or inhibited in different diseases. It is a promising therapeutic research field to inhibit or replace microRNAs.^[14]^ One study found that miRNA-424-5p regulated ferroptosis by targeting Acyl-CoA Synthetase Long Chain Family Member 4 in OC cells and indicated a potential therapeutic target for ovarian cancer.^[15]^ Wu et al^[16]^ found that the downregulated microRNA-1301-3p inhabited lung carcinoma cell proliferation and migration and has a strong negative correlation with Polymerase I and transcript release factor. Another study also indicated that the expression level of miR-200-b control PD-L1 expression in lung carcinoma cells. Furthermore, it may function as a potential biomarker for PD-L1 expression in lung cancer patients.^[17]^ These studies indicated that microRNAs may be related to the progression of cancer, and their mechanisms may take part in the pathogenesis of tumor via controlling cancer-associated genes.

In our study, data obtained from bioinformatics databases were utilized for selecting differentially expressed miRNAs (DEMs) between non-metastasis tissues and lung metastasis OS tissues. By utilizing FunRich, km-plot software, and performing an immunohistochemistry (IHC) experiment, genes related to the development of OS with lung metastasis and subsequent pathways were identified.

## 2. Methods

### 2.1. Microarray data

The RNA-seq data of OS samples and corresponding normal ovarian tissues were retrieved from the Gene Expression Omnibus (GEO) dataset (https://www.ncbi.nlm.nih.gov/geo/). The datasets of GSE38698 and GSE85537 were download and divided into 2 groups.

### 2.2. Differently expressed miRNAs analysis

GEO2R is software for differential analysis of expression microarray based on the GEO database. Limma R package was used to identify differentially expressed genes (DEGs) in the construction cohort. The screening standards of DEGs for functional enrichment analysis were |log2FC|> 1 and FDR < 0.05.

### 2.3. Gene ontology and pathway enrichment analysis

Transcription factors, Kyoto Encyclopedia of Genes and Genomes (KEGG) and Gene Ontology (GO) enrichment analyses of the DEGs were performed by using R clusterProfiler package, including package of “GOplot,” “ggplot2,” “stringi,” “colorspace” and “digest.” Then, the pathway and process enrichment analyses were carried out by using Cytoscape.

### 2.4. MicroRNA-mRNA regulatory network

At present, there are 2 generally recognized miRNA mechanisms: miRNA mediated mRNA translation inhibition and miRNA mediated mRNA specific cleavage. In addition, researchers also found that miRNA may have other regulatory mechanisms, such as regulating the localization or stability of target mRNA, or acting on target molecules other than mRNA, such as complementary binding with regulatory non coding RNA or even miRNA, or competing with other RNAs to bind proteins to achieve its regulatory function. DEMs were entered into the FunRich software to achieve target genes. Furthermore, GSE85537 was researched by utilized R software. Based the prediction conclusions of target mRNAs in FunRich software and the differentially expressed mRNAs of GSE85537, the microRNA-mRNA network was constructed.

### 2.5. Survival analysis of DEGs

Kaplan–Meier plotter can assess the impact of 54K (mRNA, miRNA, protein) on the survival rate of 21 types of cancer [including breast cancer (n = 6234), ovarian cancer (n = 2190), lung cancer (n = 3452), and gastric cancer (n = 1440)]. The sources of Kaplan–Meier plotter database include GEO, and The Cancer Genome Atlas (TCGA). The main purpose of the tool is the discovery and validation of survival biomarkers based on meta-analysis. By uploading the DEGs we identified, corresponding survival curves were obtained.

### 2.6. Correlation between gene expression and clinical samples

The data of 101 patients with OS were downloaded in the TCGA database. ARHGEF3, BSN, PQLC3, and Solute Carrier Family 7 Member 8 (SLC7A8) mRNA level, Clinical sample data and general characteristics of patients with OS were obtained.

## 3. Immunohistochemical staining

HPA database was utilized for further study. Two consecutive 5-m sections were taken from each formalin-fixed paraffin-embedded block and mounted on the glass slide treated with aminoalkyl silane, dewaxing with xylene, passing through graded alcohol, and then continue rinsing in deionized water and phosphate-buffered saline. It was blocked by 3% non-immune horse serum. The sections were incubated with broad-spectrum anti-cytokeratin AE1/ 3 (Dako, Santa Barbara, CA) in 1:50 dilution overnight at room temperature. After washing twice in buffer, an appropriate biotinylated secondary antibody was applied for 30 minutes. After 2 more washes in the buffer, appropriate biotinylated secondary antibodies were applied for 30 minutes. The sections were developed under the microscope in Tris HCl buffer (pH 7.4) and 0.03% hydrogen peroxide for 20 minutes. Application of light Mayer hematoxylin. The expressions of SLC7A8 and β-catenin were detected by the Chi-square test or Fisher exact test. SPSS software was utilized for statistical analysis. *P* value < .05 was considered statistically significant.

### 3.1. Real-time quantitative polymerase chain reaction

The expression amount of molecules is downloaded from TCGA database. The amplification conditions are 95°C for 15 seconds, then 40 cycles, 95°C for 5 seconds, 60°C for the 30 seconds. Primer sequences of SLC7A8 were as follows: primer F, 5′-CTCCACTGGA AAAAAGGTAGCA-3′, primer R, 5′-TGGTGAATGA AGCCACATCTG-3′. Primer sequences of microRNA-506 were as follows: primer F, 5′-GGGTATT GAGGAAGGTGTT-3′. primer R, 5′-CAGTGCGT GTCGTGGAGT-3′. Using glyceraldehyde-3-phosphate dehydrogenase as endogenous control, the recorded data were analyzed and processed by 2−ΔΔCt.

## 4. Results

### 4.1. Identification of DEGs

**Figure 1.**
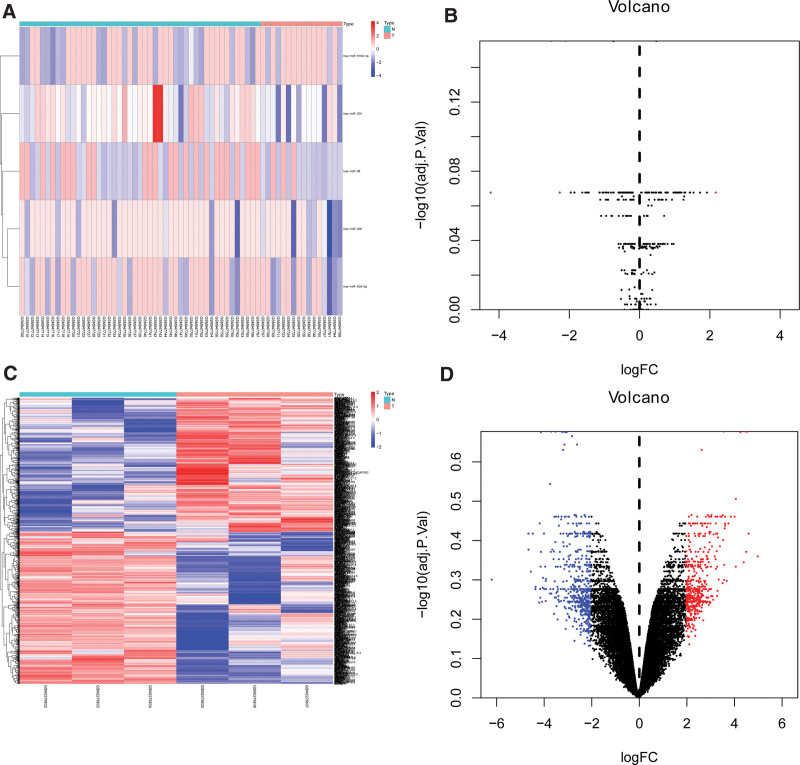
Heat map and Volcano map of DEGs of GSE38698 and GSE85537. (A) Heat map of DEGs in GSE38698. (B) Volcano map of DEGs in GSE38698. (C) Heat map of DEGs in GSE85537. (D) Volcano map of DEGs in GSE85537. Red dots represent up-regulated genes and blue dots represent down-regulated genes. DEGs = differentially expressed genes.

We used GEO2R to analysis the DEMs and DEGs from the GSE38698 and GSE85537. According to the cutoff criteria, 5 DEMs such as hsa-miRNA-205, hsa-miRNA-506, hsa-miRNA-98, hsa-miRNA-509-5p, hsa-miRNA-518d-3p and 991 DEGs were identified (Fig. 1).

### 4.2. GO enrichment analysis

**Figure 2.**
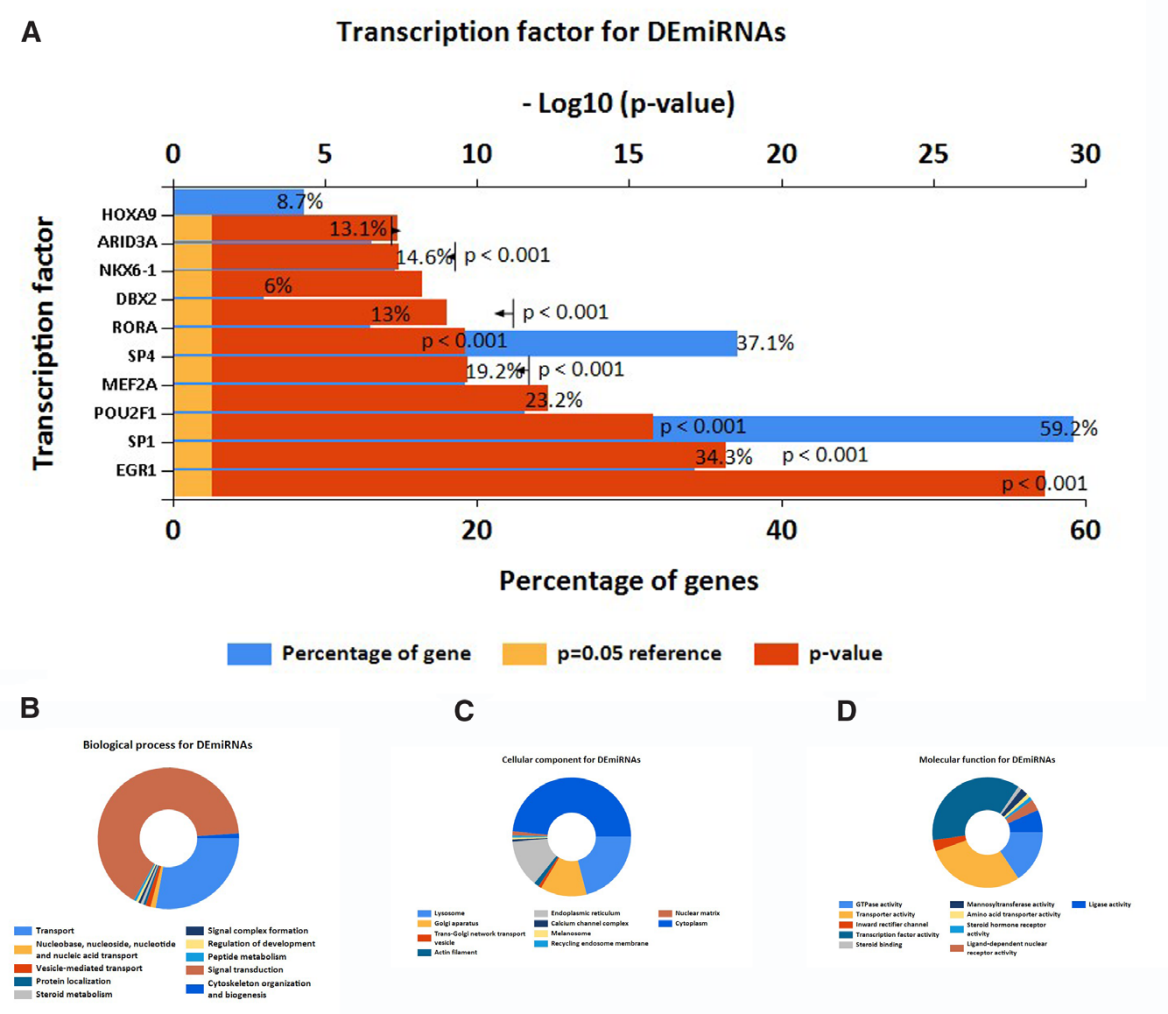
Gene ontology enrichment. (A) Transcription factors enrichment analysis of DEMs by FunRich software. (B) Biological process, (C) cellular component, and (D) molecular function enrichment analysis of the DEMs. DEMs = differentially expressed miRNAs.

**Figure 3.**
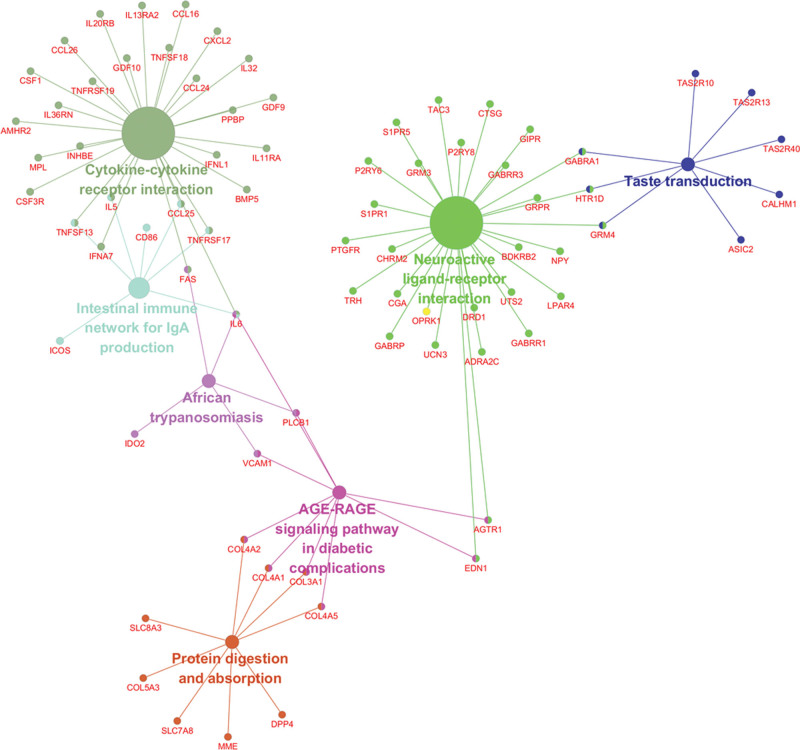
KEGG pathway enrichment analysis of DEMs. DEMs = differentially expressed miRNAs, KEGG = Kyoto Encyclopedia of Genes and Genomes.

By utilizing FunRich and R software, we performed transcription factors enrichment analysis (Fig. 2A). As for GO function analysis, 3 GO were selected: molecular function, cellular component, and biological process. Expression analysis showed that DEGs had most uniquely enriched terms for the Vesicle-mediated transport, Protein localization, Steroid metabolism, Signal complex formation, Golgi apparatus, Trans-Golgi network transport vesicle, Actin filament, Endoplasmic reticulum, Inward rectifier channel, Transcription factor activity, Steroid binding, and Mannosyltransferase activity (Fig. 2B–D). Besides, DEMs were mainly enriched in 6 pathways: cytokine-cytokine receptor interaction, neuroactive ligand-receptor interaction, intestinal immune network for IgA production, taste transduction, AGE-RAGE signaling pathway in diabetic complications, protein digestion and absorption, and African trypanosomiasis (Fig. 3).

### 4.3. MiRNA-mRNA network

**Figure 4.**
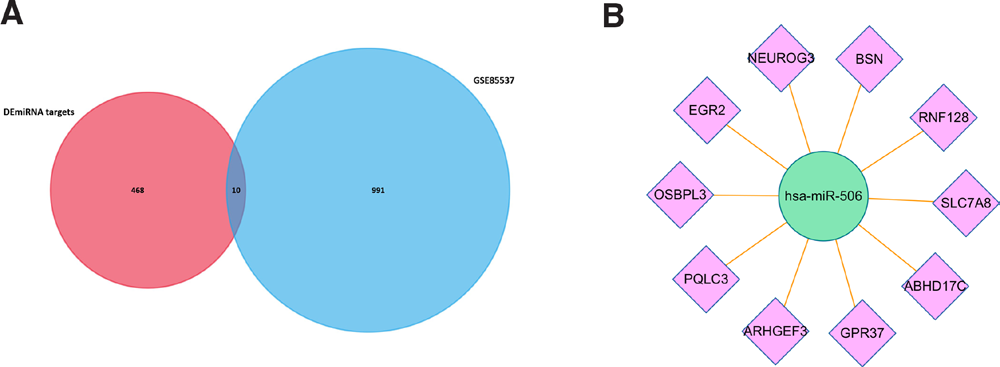
MiRNAs and their potential target mRNAs. (A) Venn diagram of GSE80201 and GSE39262. (B) Identified target mRNAs and miRNA-mRNA regulatory network. miRNA = microRNAs.

According to FunRich software, 468 mRNAs were obtained and 10 of them had different expression level in GSE27651 (ABHD17C, ARHGEF3, BSN, EGR2, GPR37, NEUROG3, OSBPL3, PQLC3, RNF128, and SLC7A8) (Fig. 4A). Based on the association between them, 10 miRNA-mRNA pairs (microRNA-506, ABHD17C, ARHGEF3, BSN, EGR2, GPR37, NEUROG3, OSBPL3, PQLC3, RNF128, and SLC7A8) were selected for next research (Fig. 4B).

### 4.4. Genes expression and their associations with OS overall survival

**Figure 5.**
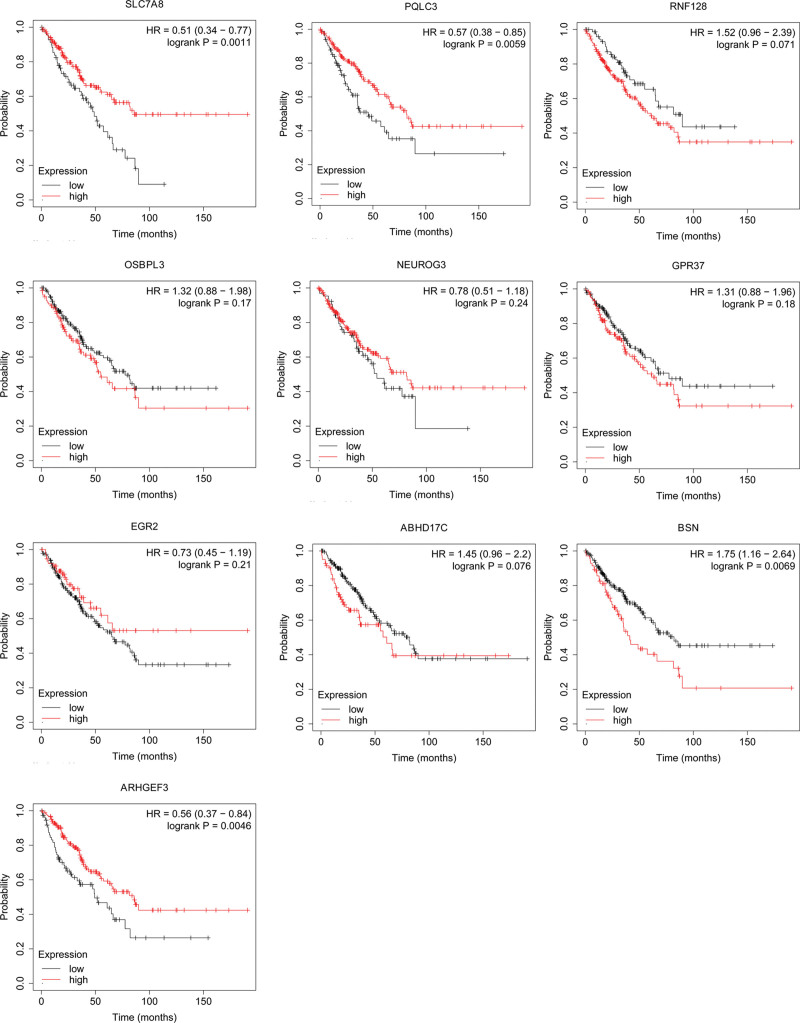
The relationship between the expression level of identified mRNAs and OS. OS = osteosarcoma.

Kaplan–Meier plot was utilized to analyze the prognosis of patients with OS. According to entering the miRNA and target gene we identified, we downloaded survival curves. The pictures showed that ARHGEF3, BSN, PQLC3, and SLC7A8 (Fig. 5) were obviously associated with the overall survival of patients with OS. However, the expression level of ABHD17C, EGR2, GPR37, NEUROG3, OSBPL3, and RNF128 may have no obvious relationship with OS.

### 4.5. Relationship between clinical characteristics and genes expression level of OS

Clinical and gene expression information of OS patients were downloaded in the TCGA database. As for ARHGEF3 and BSN, the univariate analysis and multivariate analysis indicated that the metastasis stage was associated with overall survival (*P* > .05). However, the high expression of ARHGEF3 mRNA and BSN mRNA, tumor location, age, gender and progression of primary site were not associated with the overall survival rate (*P* > .05) (Tables 1 and 2). In addition, we also studied the relationship between the expression of SLC7A8 in OS and clinical characteristics. Univariate analysis showed that the metastasis period and expression level of SLC7A8 were associated with the overall survival rate of OS (*P* < .01). However, tumor location, age, gender, and primary site progression were not associated with overall survival (*P* > .05). Multivariate analysis also showed that SLC7A8 expression level and metastasis stage was an independent risk factor for overall survival in OS (*P* < .01) (Tables 3 and 4).

**Table 1:** Univariate and multivariate Cox regression were used to analyze the relationship between the expression level of ARHGEF3 and the overall survival rate.

Characteristics	Total (N)	Univariate analysis		Multivariate analysis	
		Hazard ratio (95% CI)	*P* value	Hazard ratio (95% CI)	*P* value
Metastasis	99				
No	75	Reference			
Yes	24	3.679 (1.964–6.892)	<.001	2.871 (1.253–6.578)	.013
Tumor region	63				
Distal	36	Reference			
Other & Proximal & Proximal & Distal	27	0.473 (0.198–1.127)	.091	0.518 (0.216–1.241)	.140
Age	99				
<18	76	Reference			
≥18	23	0.732 (0.325–1.653)	.454		
ARHGEF3	99				
Low	50	Reference			
High	49	1.296 (0.695–2.416)	.415		
Primary site progression	50				
No	31	Reference			
Yes	19	1.769 (0.864–3.626)	.119		

**Table 2:** Univariate and multivariate Cox regression were used to analyze the relationship between the expression level of BSN and the overall survival rate.

Characteristics	Total (N)	Univariate analysis		Multivariate analysis	
		Hazard ratio (95% CI)	*P* value	Hazard ratio (95% CI)	*P* value
Metastasis	99				
No	75	Reference			
Yes	24	3.679 (1.964–6.892)	<.001	2.871 (1.253–6.578)	.013
Tumor region	63				
Distal	36	Reference			
Other & Proximal & Proximal & Distal	27	0.473 (0.198–1.127)	.091	0.518 (0.216–1.241)	.140
Age	99				
<18	76	Reference			
≥18	23	0.732 (0.325–1.653)	.454		
BSN	99				
Low	50	Reference			
High	49	0.750 (0.406–1.387)	.359		
Primary site progression	50				
No	31	Reference			
Yes	19	1.769 (0.864–3.626)	.119		

**Table 3:** Univariate and multivariate Cox regression were used to analyze the relationship between the expression level of SLC7A8 and the overall survival rate.

Characteristics	Total (N)	Univariate analysis		Multivariate analysis	
		Hazard ratio (95% CI)	*P* value	Hazard ratio (95% CI)	*P* value
Metastasis	99				
No	75	Reference			
Yes	24	3.679 (1.964–6.892)	<.001	4.781 (1.736–13.171)	.002
Tumor region	63				
Distal	36	Reference			
Other & Proximal & Proximal & Distal	27	0.473 (0.198–1.127)	.091	0.615 (0.253–1.494)	.283
Age	99				
<18	76	Reference			
≥18	23	0.732 (0.325–1.653)	.454		
SLC7A8	99				
Low	39	Reference			
High	60	0.464 (0.302–0.952)	.032	0.400 (0.154–0.939)	.040
Primary site progression	50				
No	31	Reference			
Yes	19	1.769 (0.864–3.626)	.119		

SLC7A8 = Solute Carrier Family 7 Member 8.

**Table 4:** Relationship between SLC7A8 expression level and clinical features of OS.

Characteristic	Low expression of SLC7A8	High expression of SLC7A8	*P*
n	50	51	
Metastasis, n (%)			
No	38 (27.7)	3 (2.9)	.021
Yes	12 (11.9)	48 (47.5)	
Tumor region, n (%)			
Distal	20 (31.2)	16 (25)	.330
Other	2 (3.1)	1 (1.6)	
Proximal	9 (14.1)	16 (25)	
Proximal & Distal	0 (0)	0 (0)	
Age, n (%)			
<18	39 (38.6)	39 (38.6)	1.000
≥18	11 (10.9)	12 (11.9)	
Gender, n (%)			
Female	21 (20.8)	20 (19.8)	.934
Male	29 (28.7)	31 (30.7)	
Race, n (%)			
American Indian or Alaska Native	0 (0)	1 (1.3)	
Asian	4 (5.3)	3 (3.9)	
Black or African American	5 (6.6)	5 (6.6)	
Native Hawaiian or other Pacific Islander	0 (0)	0 (0)	
White	27 (35.5)	31 (40.8)	
Ethnicity, n (%)			
Hispanic or Latino	9 (12.5)	2 (2.8)	.062
Not Hispanic or Latino	28 (38.9)	33 (45.8)	
Age, meidan (IQR)	14.65 (12.35, 17.69)	15.18 (12.52, 17.8)	.552

IQR= interquartile range, OS = osteosarcoma, SLC7A8 = Solute Carrier Family 7 Member 8.

### 4.6. Verification of potential biomarker expression by IHC and quantitative real-time PCR

**Figure 6.**
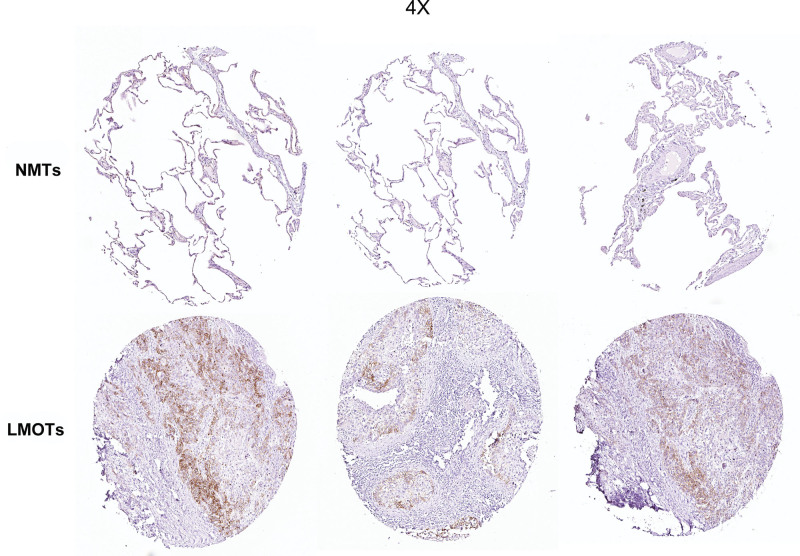
SLC7A8 expression in NMTs and LMOTs. LMOTs = lung metastasis OS tissues, NMTs = non-metastasis tissues, SLC7A8 = Solute Carrier Family 7 Member 8.

**Figure 7.**
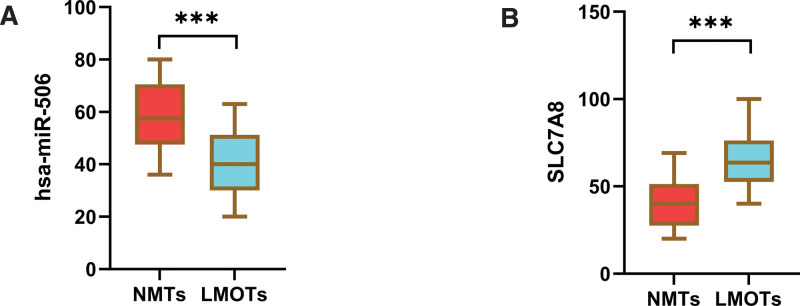
The expression level of hsa-miR-506 and SLC7A8 in NMTs and LMOTs. (A) Validation of hsa-miR-506 (***P < .001). (B) Validation of SLC7A8 (***P < .001). LMOTs = lung metastasis OS tissues, NMTs = non-metastasis tissues, SLC7A8 = Solute Carrier Family 7 Member 8.

To further assess the expression of SLC7A8 and hsa-miR-506, a total of 20 pairs of non-metastasis tissues (NMTs) and lung metastasis OS tissues (LMOTs) were enrolled as a validation cohort. The expression level of genes was detected by IHC. The staining intensity of SLC7A8 was divided into 0, 1, 2 or 3, corresponding to colorless, light yellow, light brown and squid ink. In addition, the percentage score is defined as: 0% to 5%, 0; 6% to 25%, 1 point; 26% to 50%, 2 points; 50% to 75%, 3 points; 76% to 100%, 4 points. The final histochemical score was calculated by multiplying the intensity score by the percentage score. The final staining scores were negative (0), low^[6]^ and strong (≥6). Statistically, the overall expression of SLC7A8 was much higher in LMOTs tissues than in the NMTs (*P* < .001) (Fig. 6). The qRT-PCR method was used to confirm the differential expression levels from participants’ samples. Consistent with the microarray data, microRNA-506 was significantly downregulated (Fig. 7A) and SLC7A8 was significantly upregulated (Fig. 7B) between 20 pairs of LMOTs and NMTs which indicated that hsa-miR-506 and SLC7A8 could be the candidate biomarkers for OS with lung metastasis.

## 5. Discussion

In 2020, there were 19.29 million new cancer cases worldwide, including 10.06 million males and 9.23 million females; In 2020, there were 9.96 million cancer deaths worldwide, including 5.53 million males and 4.43 million females.^[18]^ In this research, GSE38698 and GSE85537 were obtained in the GEO database. 5 DEMs and 991 DEGs were identified. For deeply understanding the process of the 5 microRNAs in OS, FunRich and R software were utilized for further study. GO and KEGG analysis indicated that DEmiRNAs were primarily associated with the Vesicle-mediated transport, Protein localization, Steroid metabolism, Signal complex formation, Golgi apparatus, Trans-Golgi network transport vesicle, Actin filament, Endoplasmic reticulum, Inward rectifier channel, Transcription factor activity, Steroid binding, and Mannosyltransferase activity. Previous studies have shown that Lysosomes and Nucleus may function as a vital role in plenty of human diseases, such as tumor, obesity, and infection.^[19–21]^ Furthermore, KEGG research showed that these genes were mainly enriched in 7 pathways which were shown to affect migration and proliferation.^[22]^ As for Cytokine-cytokine receptor interaction, cancer cells release various cytokines and growth factors to the surroundings, and recruit and reprogram many other types of cells to establish the tumor microenvironment, including tumor cells, stromal fibroblasts, endothelial cells and infiltrating leukocytes, such as macrophages, as well as T lymphocytes and tumor associated dendritic cells in the development of cancer. In addition, eosinophils, granulocytes, natural killer cells and B cells are all present in some types of tumors.^[23,24]^ IL-12 is the production of IFN by T cells and natural killer cells γ Necessary conditions for. During the physiological immune response, the expression of IL-12 increased. In many cancer patients, the expression of IL-12 decreases in this process, especially in advanced, more advanced cancers, such as astrocytoma, glioblastoma, renal cell carcinoma, head and neck squamous cell carcinoma, malignant melanoma, colorectal cancer and so on. With the development of gastric cancer and colorectal cancer, the production of IL-12 by stimulated peripheral blood monocytes decreased significantly.^[25,26]^ The regulated network was performed according to Cytoscape. 5 miRNAs (hsa-miRNA-205, hsa-miRNA-506, hsa-miRNA-98, hsa-miRNA-509-5p, hsa-miRNA-518d-3p) were selected for further research. After that, 991 target genes were achieved and 10 of them showed different expression level in GSE39262 (ABHD17C, ARHGEF3, BSN, EGR2, GPR37, NEUROG3, OSBPL3, PQLC3, RNF128, and SLC7A8). MiR-506 is one of the miRNAs closely related to tumor proliferation and metastasis. More and more studies have shown that the antitumor effect of miR-506 is achieved by inhibiting cell proliferation, inducing cell cycle arrest, increasing cancer cell apoptosis, and sensitivity to chemotherapy.^[27]^ It was reported that microRNA-506 inhibited OS,^[28]^ neural stem cell,^[29]^ esophageal squamous cell cancer,^[30]^ and non-small lung carcinoma^[31]^ progression. Solute transporter 7 (SLC7A8) family member 8 is a sodium independent amino acid exchanger (antiports), which can transport large and small neutral amino acids, such as alanine, serine, threonine, cysteine, phenylalanine, tyrosine, leucine, and glutamine. It needs to be isomerized with the heavy chain of SLC3A2 to be correctly located in the plasma membrane.^[32]^ SLC7A8 lacks research to clarify its role in human cancer prognosis. Statistics from Oncomine showed that SLC7A8 is significantly up-regulated in several carcinomas, including breast carcinoma, colorectal carcinoma, head and neck carcinoma, leukemia, lymphoma, and melanoma.^[33]^ However, this was only verified at the mRNA level of some breast tumors and melanoma cell lines.^[34]^ However, how SLC7A8 promotes lung metastasis of OS has not been reported.

Many studies have shown that the abnormal expression of miRNAs is caused by gene aberrations (including genetic and epigenetic changes) of many cancer types, and plays a role in the occurrence and development of cancer through the imbalance of target gene expression. Therefore, many miRNAs and their target genes are closely related to the pathogenesis of tumor, including cell proliferation, cell survival and cell invasion. Our study indicated that plenty of DEGs and DEMs were taken part in the process of OS by some pathways and had prognostic value. Therefore, suppression of SLC7A8 and upregulated miR-506 may have latent remedy worth in OS patients.

## 6. Conclusion

Our study indicated some reasons for the procession of OS with lung metastasis. Plenty of DEMs and DEGs were selected between LMOTs and NMTs. Besides, miR-506 and SLC7A8 were selected as latent biomarkers of LMOTs. quantitative real-time PCR and IHC results indicated that microRNA-506 and SLC7A8 were differentially expressed between LMOTs and NMTs. However, we need more cell experiments to prove it.

## Author contributions

**Conceptualization:** Fanjian Meng.

**Data curation:** Lulu Wang.

**Formal analysis:** Jinpeng Chen.

**Funding acquisition:** Xinghua Wang.

**Investigation:** Gaochen Wu, Yiqi Miu.

**Project administration:** Guangyu Gao.
